# Comparison of four different colorimetric and fluorometric cytotoxicity assays in a zebrafish liver cell line

**DOI:** 10.1186/1471-2210-8-8

**Published:** 2008-05-30

**Authors:** Stephanie K Bopp, Teresa Lettieri

**Affiliations:** 1 European Commission – Joint Research Centre, Institute for Environment and Sustainability, Rural, Water, and Ecosystem Resources Unit, Via E. Fermi 2749, 21027 Ispra (VA), Italy

## Abstract

**Background:**

A broad spectrum of cytotoxicity assays is currently used in the fields of (eco)toxicology and pharmacology. To choose an appropriate assay, different parameters like test compounds, detection mechanism, specificity, and sensitivity have to be considered. Furthermore, tissue or cell line can influence test performance. For zebrafish (*Danio rerio*), as emerging model organism, cell lines are now increasingly used, but few studies examined cytotoxicity in these cell systems. Therefore, we compared four cytotoxicity assays in the zebrafish liver cell line, ZFL, to test four differently acting model compounds. The tests comprised two colorimetric assays (MTT assay using 3-[4,5-dimethylthiazol-2-yl]-2,5-diphenyl tetrazolium bromide, and the LDH assay detecting lactate dehydrogenase activity) and two fluorometric assays (alamarBlue^® ^using resazurin, and CFDA-AM based on 5-carboxyfluorescein diacetate acetoxymethyl ester). Model compounds were the pharmaceutical Tamoxifen, its metabolite 4-Hydroxy-Tamoxifen, the fungicide Flusilazole and the polycyclic aromatic hydrocarbon Benzo[a]pyrene.

**Results:**

All four assays performed well in the ZFL cells and led to reproducible dose-response curves for all test compounds. Effective concentrations causing 10% or 50% loss of cell viability (EC_10 _and EC_50 _values) varied by a maximum factor of 7.0 for the EC_10 _values and a maximum factor of 1.8 for the EC_50 _values. The EC values were not statistically different between the four assays, which is due to the assessed unspecific effects of the compounds. However, most often, the MTT assay and LDH assay showed the highest and lowest EC values, respectively. Nevertheless, the LDH assay showed the highest intra- and inter-assay variabilities and the lowest signal-to-noise ratios. In contrast to MTT, the other three assays have the advantage of being non-destructive, easy to handle, and less time consuming. Furthermore, AB and CFDA-AM can be combined on the same set of cells without damaging the cells, allowing later on their use for the investigation of other endpoints.

**Conclusion:**

We recommend the alamarBlue and CFDA-AM assays for cytotoxicity assessment in ZFL cells, which can be applied either singly or combined.

## Background

The detection of cell viability is crucial in many biological fields, e.g. in toxicology, in pharmacology (drug development), as well as in ecotoxicology for the assessment of toxic effects elicited by chemicals, drugs or contaminated environmental samples, respectively. The need for reliable, easy to handle, and fast cytotoxicity tests led to the development of several assays which are now routinely used and available to detect cytotoxic effects in *in vitro *cellular systems.

One of the mostly used cytotoxicity or cell proliferation assays is the MTT assay, which is a quantitative colorimetric assay. In this assay, the yellow tetrazolium salt MTT (3-[4,5-dimethylthiazol-2-yl]-2,5-diphenyl tetrazolium bromide) is reduced by living cells to blue formazan crystals, which must be solubilized in a solvent, such as iso-propanol [1]. It was assumed that the tetrazolium salt is reduced in mitochondrial processes at two sites of the respiratory chain [2], but later on it was shown, that also microsomal and cytosolic fractions are involved in the formation of formazan [3,4]. The MTT assay appeared to be a sensitive test which shows linearity over a broad range of cell densities [1,5].

Another commonly used assay is the lactate dehydrogenase (LDH) assay. It is a colorimetric assay based on the detection of LDH activity which is released from the cytosol of damaged or lysed cells. Thus the evaluation of cytotoxicity is based on plasma membrane integrity [6,7]. It is a non-destructive measurement technique since it can be performed using the culture medium. Also in this assay, a tetrazolium salt, INT (2-[4-iodophenyl]-3-[4-nitrophenyl]-5-phenyltetrazolium chloride), is involved in the colorimetric reaction. Coupled to the oxidation of lactate to pyruvate, INT is transformed to formazan which can be detected spectrophotometrically. Another possibility is to measure directly the formation of NADH by absorption [8]. Since LDH is a relatively stable enzyme [7], the culture medium can also be stored before performing the measurement [8].

A further assay, working similarly as MTT, is the alamarBlue (AB) assay, which measures cellular metabolic activity. The AB assay is based on the conversion of the blue non-fluorescent dye resazurin, which is converted by mitochondrial and other enzymes to the pink fluorescent resorufin [4,9]. Resorufin can be detected spectrophotometrically or fluorometrically [10]. Since both, the oxidized substrate resazurin and the reduced product resorufin, are water soluble, they can freely diffuse along concentration gradients. In addition, AB shows no cytotoxic effects and the tested cells do not need to be destroyed, thus enabling to perform several tests or kinetic measurements on the same set of cells [9,11,12].

CFDA-AM (5-carboxyfluorescein diacetate acetoxymethyl ester) is another fluorogenic dye, which is used for cytotoxicity studies, indicating plasma membrane integrity. The dye CFDA-AM is a non-toxic esterase substrate that can be converted by nonspecific esterases of living cells from a membrane permeable, nonpolar, nonfluorescent substance to a polar, fluorescent dye, carboxyfluorescein (CF). The conversion to CF by the cells indicates the integrity of the plasma membrane, since only an intact membrane can maintain the cytoplasmic milieu which is needed to support esterase activity [10]. AB and CFDA-AM were already shown to be applicable in parallel on the same set of cells, since both are non-toxic to cells, require similar incubation times, and can be detected at different wavelengths without interferences [10-14].

The performance of cytotoxicity assays strongly depends on tissue or cell line under investigation. Therefore, before choosing a cytotoxicity assay for a new application, different methods should be compared. Along these lines, the current study had two major aims: firstly, to test the compatibility of these assays with the zebrafish (*Danio rerio*) liver cell line ZFL [15] and to set up the optimal test conditions. Secondly, to identify which test or test combination could deliver most useful information for cytotoxicity studies in ZFL cells since, to our knowledge, there is not much experience available on cytotoxicity assays in the ZFL cell line. Few data have been published on MTT and AB, which were applied each once to assess cell viability in presence of arsenite and heavy metals, respectively [16,17]. For evaluating the four above mentioned assays, we investigated four model compounds from three different substance groups: the polycyclic aromatic hydrocarbon Benzo[a]pyrene (BaP), the triazole fungicide Flusilazole (Flus), the pharmaceutical Tamoxifen (Tam) and its active metabolite 4-Hydroxy-Tamoxifen (4-OHT).

## Results

In the first step, all assays were established in the ZFL cell line investigating different influencing parameters, such as cell number per well or medium constitution, before testing the model compounds to compare the assay performances.

**Figure 1:**
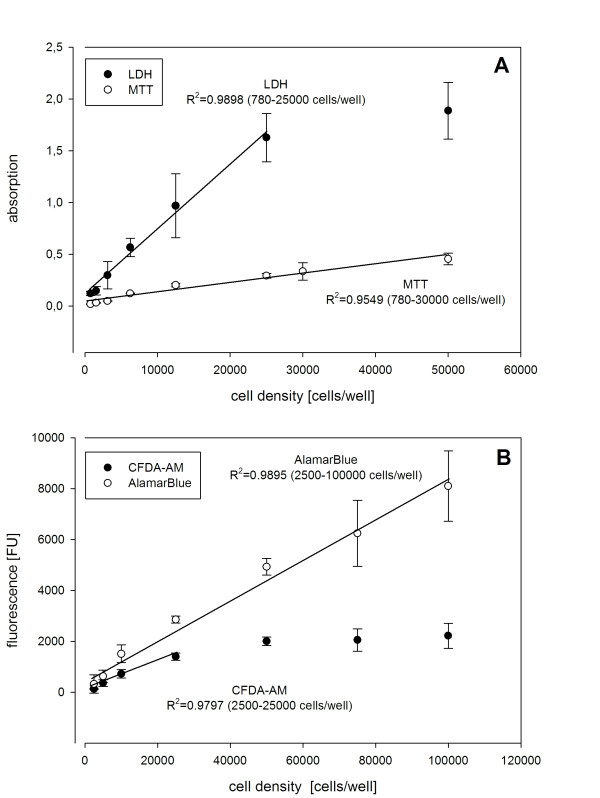
**Effects of different cell densities**. Effects of different cell densities were studied in the four cytotoxicity assays and the linear range determined by linear regression. For all assays 25,000 cells/well was selected to be used as standard cell number in the cytotoxicity tests, which was included in the linear range for all assays. Panel A shows the results for the LDH and MTT assays with the Y-axis indicating absorption with respect to the reference wavelengths, i.e. abs_590 _with reference at 655 nm for LDH and abs_570 _with reference at 680 nm for MTT. Panel B shows linearity for the AB and CFDA-AM assays, with the Y-axis indicating the fluorescent units [FU] detected at 530 nm excitation/590 nm emission for AB and 485 nm/535 nm for CFDA, respectively.

For the MTT assay, a commercially available kit was used. Therefore, only the parameters cell density and incubation time were examined. Incubation of the cells with MTT for 2, 4, or 6 h led to no significant differences in absorption. Therefore, a four hour incubation time was applied as suggested in the manufacturer's protocol. In addition, different cell densities were used to optimize test conditions. A linear relationship between cell number and absorption could be established for up to 50,000 cells/well (R^2 ^= 0.95) (Figure 1A). However, since cell densities of ≤12,500 cells/well led to relatively low signals, 25,000 cells/well were used in optimized standard tests conditions.

The effect of different cell densities was also investigated for the LDH assay for which we used a commercially available kit. Since the LDH assay detects lysed and not viable cells, the cells need to be destroyed for these investigations to optimize test conditions. Therefore, the positive control Triton X-100 was used, at the concentration of 2% to completely lyse the cells. A linear relationship between cell number and absorption was observed (R^2 ^= 0.99) and 25,000 cells/well were used as standard condition (Figure 1A). Furthermore, the effect of FBS on the LDH activity was investigated, and we observed that lowering the amount of FBS to 1% in the medium greatly reduced background values (see Materials and Methods). Consequently, 1% FBS was used in the standard test condition.

The AB and CFDA-AM assay were established directly in combination, since many examples in the literature showed that there were no interferences of the two dyes in other cellular systems, such as e.g. in the rainbow trout gill cell line RTgill-W1 [10,11] as well as in primary hepatocytes from mouse and rainbow trout and in the cell lines HepG2 and RTL-W1 [18]. The simplified Earle's-G medium [19], which was used to replace the standard ZFL culture medium during measurement, was shown to have no negative effect on cell viability. Furthermore, test performance was examined using different cell densities and a linear relationship between fluorescence and cell number was found for cell densities up to 25,000 cells/well with a correlation coefficient R^2 ^of 0.99 for AB and 0.98 for CFDA-AM, respectively (Figure 1B).

**Figure 2:**
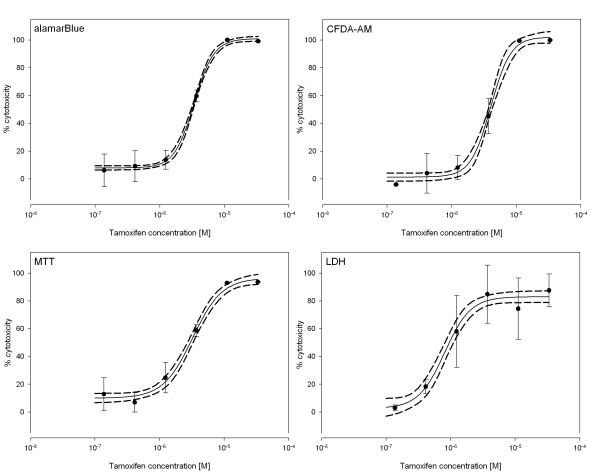
**Cytotoxic effects of Tamoxifen**. Dose-response curves for cell viability assessment in ZFL cells exposed to Tamoxifen using the four different assays. Closed circles represent the mean of the measured triplicate wells with vertical lines indicating the standard deviation within one single experiment. The continuous line represents the fitted dose-response curve while dashed lines indicate the 95% confidence interval.

**Figure 3:**
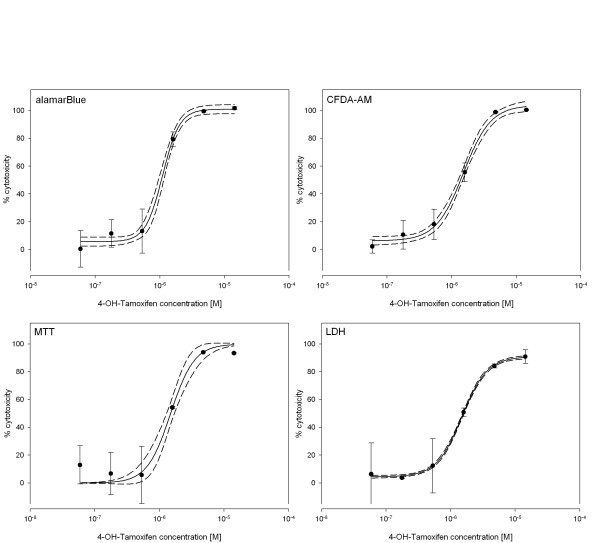
**Cytotoxic effects of 4-Hydroxy-Tamoxifen**. Dose-response curves for cell viability assessment in ZFL cells exposed to 4-OH-Tamoxifen using the four different assays. Closed circles represent the mean of the measured triplicate wells with vertical lines indicating the standard deviation within one single experiment. The continuous line represents the fitted dose-response curve while dashed lines indicate the 95% confidence interval.

**Figure 4:**
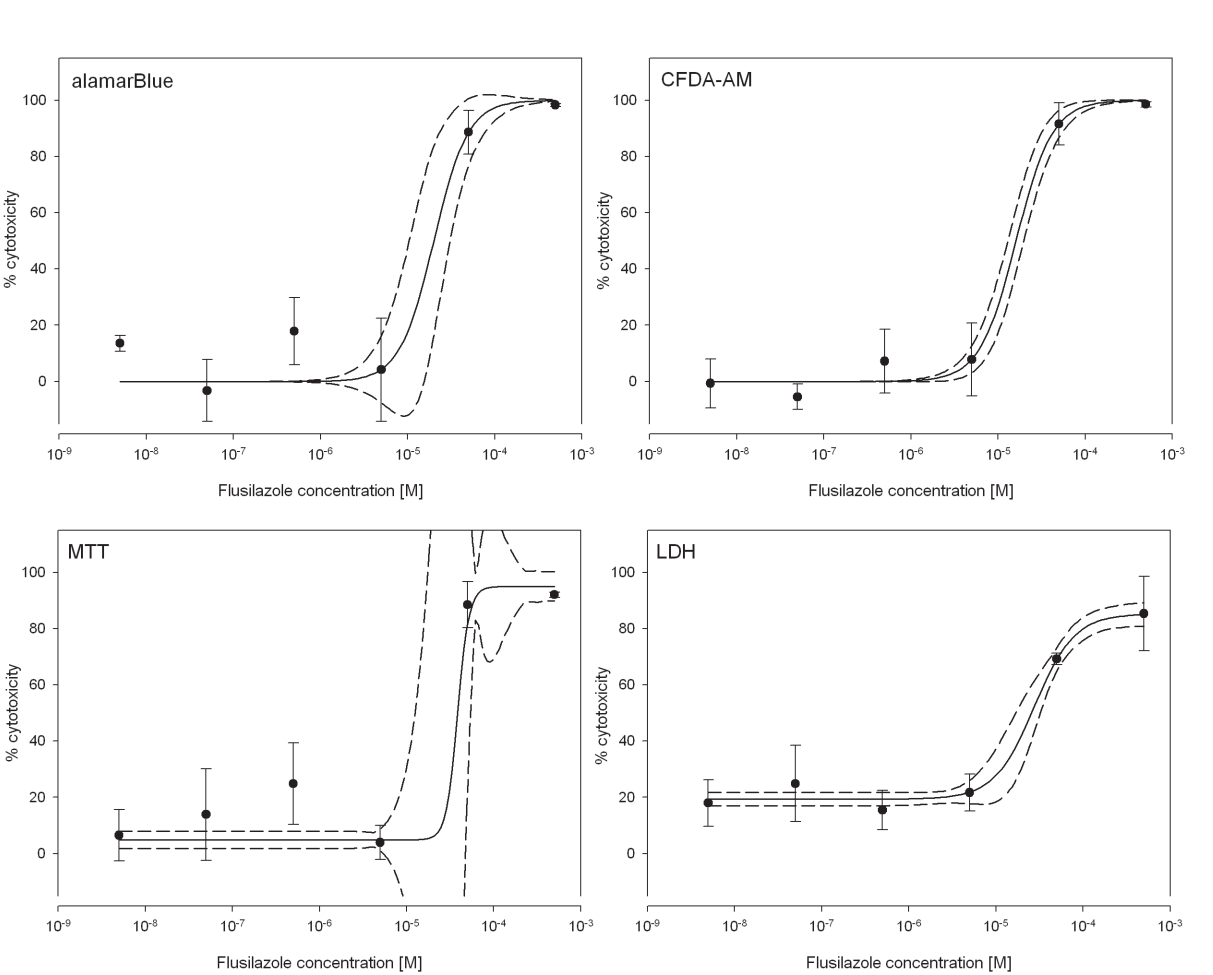
**Cytotoxic effects of Flusilazole**. Dose-response curves for cell viability assessment in ZFL cells exposed to Flusilazole using the four different assays. Closed circles represent the mean of the measured triplicate wells with vertical lines indicating the standard deviation within one single experiment. The continuous line represents the fitted dose-response curve while dashed lines indicate the 95% confidence interval.

Applying the established assays to test the four model compounds in ZFL cells, all four assays performed well and led to reproducible dose-response curves (Fig. 2, 3, 4). We determined effective concentrations causing a 10% or 50% inhibition of cell viability for each compound except for BaP. Indeed, for BaP cytotoxic effects were not higher than 37% even at concentrations either close to or above the aqueous solubility (data not shown). Therefore, effective concentrations could not be calculated for none of the assays for BaP.

For the three other test compounds, effective concentrations were calculated (Table 1). For exposure of ZFL cells to Tamoxifen, the LDH assay was the most sensitive, whereas CFDA-AM showed the highest EC_10 _and MTT the highest EC_50 _value. For 4-OHT experiments, LDH showed the lowest EC_10_, while CFDA-AM showed the lowest EC_50 _value, and the MTT assay was the least sensitive. For Flusilazole, the AB assay led to the highest EC_10 _and EC_50 _values. The LDH assay and CFDA-AM assay were similar to the 4-OHT experiment, in terms of lowest EC_10 _and EC_50 _value, respectively. However, the observed differences between the EC values from the different assays for each test compound were relatively small with a maximum factor of 1.8 occurring for EC_50 _values and a maximum factor of 7.0 for EC_10 _values. The deviations between the different assay EC values were not statistically significant for none of the compounds (One way ANOVA, Tukey's test, p < 0.05).

**Table 1:** 10% and 50% effective concentrations (EC) for cytotoxicity in ZFL cells.

	Tamoxifen		4-OH-Tamoxifen		Flusilazole	
			
	EC_10 _[μM]	EC_50 _[μM]	EC_10 _[μM]	EC_50 _[μM]	EC_10 _[μM]	EC_50 _[μM]
LDH	0.61 ± 0.67	1.88 ± 1.01	0.73 ± 0.55	1.62 ± 0.01	2.28 ± 3.12	17.37 ± 14.53
MTT	1.39 ± 0.77	3.46 ± 0.70	1.49 ± 0.82	1.78 ± 0.40	11.29 ± 5.88	18.37 ± 14.11
AB	1.37 ± 0.33	3.12 ± 0.25	0.79 ± 0.40	1.17 ± 0.24	16.13 ± 11.44	26.40 ± 7.30
CFDA-AM	2.52 ± 2.14	3.35 ± 0.75	1.08 ± 0.62	1.73 ± 0.67	6.49 ± 1.08	16.68 ± 4.76

Effective concentrations (EC) causing 10 or 50% loss of cell viability were determined from a four parameter logistic equation fitted to the experimental data for three individual experiments. EC values shown in the table are mean values from three experiments ± standard deviation. No EC values could be determined for BaP since dose-response curves were incomplete (highest concentrations which were already above aqueous solubility caused 37% loss in cell viability at most). EC values were not significantly different from each other (One way ANOVA, Tukey's test, p < 0.05).

For comparison of the four cytotoxicity assays, the parameters intra- and inter-assay variability, as well as signal-to-noise ratio were determined (Table 2). The intra-assay variabilities were measured using triplicate samples within each experiment and are illustrated in Figure 2, 3, 4 as error bars representing the standard deviation. The coefficients of variance (CV) were calculated at high and low effect levels (Table 2). The AB, CFDA-AM, and MTT assays showed low variability at higher signals, i.e. at higher cytotoxicities, and higher variability at low cytotoxicities, whereas the LDH assay showed overall high intra-assay variability. The inter-assay variability was derived from the EC_10 _and EC_50 _values calculated for each of the three individual experiments (Table 1 and 2). Inter-assay variability was higher for all assays at the EC_10 _level than at the EC_50 _level. The LDH assay showed CV values at both levels which were approximately the double of the CV values for the other three assays, thus indicating a much higher inter-assay variability. Furthermore, the signal-to-noise ratios were calculated using the signals from wells without cells as background noise values. For the AB, CFDA-AM, and the MTT assay, with all of them detecting live cells, low ratios close to background level were observed at high compound concentrations causing 80–100% loss of cell viability, whereas ratios of 28, 7, and 22 were observed for the AB, CFDA-AM, and MTT assay, respectively, at the lowest compound concentrations (0–20% cytotoxicity). For the LDH assay, which detects released enzyme activity from lysed cells, signals were close to background level at low compound concentrations. Maximum ratios for LDH were observed at the higher compound concentrations with a ratio of 2.2.

**Table 2:** Quality parameters intra- and inter-assay variability and signal-to-noise ratio for the four cytotoxicity assays.

	Intra-assay variability (CV [%])		Inter-assay variability (CV [%])		Signal-to-noise ratio	
			
	0–20% cytotoxicity	80–100% cytotoxicity	10% cytotoxicity (EC_10_)	50% cytotoxicity (EC_50_)	0–20% cytotoxicity	80–100% cytotoxicity
LDH	62	15.5	110	54	1.4	2.2
MTT	118	0.6	55	23	21.6	2.1
AB	130	0.8	51	21	5.7	1.0
CFDA-AM	102	0.3	57	29	22.8	1.1

Variability of cytotoxicity assays based on experiments assessing Tam, 4-OHT, and Flus toxicity. Intra-assay variability was calculated based on triplicate wells assessed within one assay and median values were calculated based on three independent experiments. Inter-assay variability was calculated as the deviation of effective concentrations based on each three independent experiments. For both, intra- and inter-assay variability, the coefficient of variance (CV [%]) is shown in the table. The signal-to-noise ratio was calculated for Tam exposure experiments by dividing the absorption or fluorescence in the wells containing untreated or treated cells by the signal in background wells without cells. This was performed for each Tam concentration and median values are shown for low (0–20%) and high (80–100%) cytotoxicity ranges.

## Discussion

Four different assays were established in the zebrafish liver cell line ZFL to detect the cytotoxicity upon exposure to the four tested compounds, Benzo[a]pyrene (BaP), Tamoxifen (Tam), its metabolite 4-Hydroxy-Tamoxifen (4-OHT) and Flusilazole (Flus). BaP is known to act as a dioxin-like compound, inducing CYP1A1 in juvenile zebrafish and its embryos, but also inducing CYP19 and vitellogenin [20-22]. The presence and inducibility of CYP1A1 in ZFL cells was shown using the dibenzodioxin TCDD [23,24] and induction of *cyp1A1 *gene expression in BaP exposed ZFL cells was confirmed [25]. BaP was reported to be metabolized via CYP1A and to form DNA adducts [26]. Tam is a widely used pharmaceutical agent for breast cancer treatment, which is biotransformed to its active metabolite 4-OHT [27]. Tam and 4-OHT have been shown to stimulate oocyte maturation for zebrafish *in vivo *[28]. Furthermore, Tam led to increased perturbations and mortality in a partial-life cycle test with zebrafish [29]. Flus is a sterol biosysnthesis inhibiting triazole fungicide, which blocks the enzyme 14-α-demethylase [30] and interferes also with sterol pathways in mammals [31,32]. However, so far no studies are reported in zebrafish, neither *in vitro *nor *in vivo*.

All the assays performed well and no significant differences were found for the EC values from the different assays for each of the substances. This might be explained by the fact that none of these compounds specifically interferes with the processes involved in the mechanism of the assays.

On the other hand, such results give the possibility to compare in an unbiased way all the assays. Based on the detected EC values, they can all be recommended for the application to ZFL cells. However, assessing the assays based on all investigated parameters, i.e. EC values, signal-to-noise ratio, inter- and intra-variability, slight differences arose among them. Indeed, the LDH assay showed often the lowest EC values, as also reported in the literature by Kemp and Brouwer (2004) [33], which might be due to the difference that it is the only one of the four tested assays detecting lysed cells, whereas the others detect live cells. On the other hand, it was the one with the lowest signal-to-noise ratios and the highest inter- and intra-assay variabilities, thus making the detection of low cytotoxic effects very difficult. Moreover, the LDH assay uncertainties increase at lower compound concentrations, which is the opposite for the other three assays. Hence, it can be summarized for the LDH assay that it has the advantage of using only a part of the culture medium, thus enabling the use of the unaffected cells for further analysis, but even if showing the lowest EC values, it entails the disadvantage of the highest variabilities.

The MTT assay showed much lower variabilities and higher signal-to-noise ratios than the LDH assay. However, the highest EC values were observed for the MTT assay and in contrast to all other three assays, the MTT assay requires the destruction of the cells for the analysis, thus making it impossible to use the cells for other investigations and additionally it is more time consuming. On the other hand it has the advantage that no washing steps are needed and reagents are added directly to the medium, preventing enhanced variability due to procedural steps.

The AB and CFDA-AM assays showed, similarly to the MTT assay, low variabilities and high signal-to-noise ratios, suggesting that these assays are more precise and robust than the LDH assay. Furthermore, as for the LDH assay, they are nondestructive assays, which can be performed within a very short time. Both can be detected spectrophotometrically or fluorometrically [10]. AB and CFDA-AM exert no toxic effects on the cells so that even after cell viability measurement cells might be used to examine other endpoints. For example, it was shown before that a combined AB/CFDA-AM assay did not affect the results of later gene expression analysis of hepatocytes from rainbow trout (*Oncorhynchus mykiss*) [12].

## Conclusion

Based on the assessed parameters and handling of the four different assays, we recommend the AB and the CFDA-AM assay for cytotoxicity assessment. Our data show that these two assays can be also easily applied in combination to ZFL cells, thus enabling the simultaneous assessment of two different endpoints.

## Methods

### Routine culture of zebrafish liver cells (ZFL)

The zebrafish (*Danio rerio*) liver cell line ZFL [15] was obtained from ATCC (Promochem). Cells were cultured in medium composed of 50% Leibovitz L-15, 35% DMEM, and 15% Ham F-12, supplemented with 15 mM HEPES, 0.15 g/L NaHCO_3_, 0.01 mg/mL insulin, 50 ng/mL epidermal growth factor (EGF), and 5% fetal bovine serum (FBS) as proposed by ATCC. Cells were cultured at 28°C in 75 cm^2 ^cell culture flasks (Stratagene). Cells were subcultured every 5–7 days. For this purpose, they were first rinsed with 1 mL of 0.25% trypsin EDTA (Gibco), and then detached with 1 mL of trypsin EDTA. Trypsination was stopped by addition of 5 mL of 10% FBS containing medium. Cells were centrifuged for 5 min at 300 g. Cell pellets were resuspended in 5% FBS containing medium and either split to new flasks or plated in 96 well flat-bottom culture plates for cytotoxicity assays.

### Exposure of ZFL cells for cytotoxicity assessment

For comparison of the four cytotoxicity assays, cells were exposed to four differently acting model compounds: the polycyclic aromatic hydrocarbon Benzo[a]pyrene (BaP; purity ≥ 97%, Fluka), the fungicide Flusilazole (Flus; purity 99.8%, Riedel-de Haën), the pharmaceutical Tamoxifen (Tam) as Tamoxifen citrate (purity ≥ 99%, Sigma) and its metabolite *trans*-4-Hydroxy-Tamoxifen (4-OHT; purity ≥ 98%, Sigma). Each compound was dissolved in dimethyl sulfoxide (DMSO) and diluted in a way that all stock solutions were 200 times concentrated compared to the final test concentrations. Concentration ranges were 30 pM-1.97 μM for BaP, 5 nM-5 μM for Flus, 5 nM-11 μM for Tam, and 0.2–43 μM for 4-OHT, which is in the range of *in vivo *studies with waterborne exposure of zebrafish for BaP, Tam, and 4-OHT [20-22,29]. For Flusilazole, no studies on fish are available.

For finding the optimal biological test conditions, in the beginning different cell densities were applied using two-fold dilution series in the range of 780–100,000 cells/well and cell viability tests were performed after 24 h. 25,000 cells/well turned out to be in the optimum range for all of the tests. Thus, cells were plated in 96 well flat-bottom plates at an initial cell density of 25,000 cells/well in 200 μL of medium and allowed to settle for 24 h. In parallel, wells containing only medium without cells were prepared and used later on for background correction. After the pre-incubation period, for each test compound and each concentration, 1 μL of the DMSO stock solutions was added so that the final DMSO content was 0.5%. All tests were performed at least three times and each time in triplicate.

### MTT assay

The MTT assay was performed using the cell proliferation kit I MTT (Roche) according to the manufacturer's protocol. In brief, after 24 h of incubation with the test compounds, 100 μL of medium were removed from each well. Then, 10 μL of the MTT solution were added and plates incubated for 4 h at 28°C. Since the manufacturer recommends incubation at 37°C and ZFL cells have to be incubated at 28°C, we tested different incubation times with MTT (2, 4, and 6 h) to establish optimized conditions. No relevant differences were observed for the different time points, so that the 4 h incubation was used. After the incubation with MTT, the solubilization solution was added and plates incubated over night. The next day, absorption of the produced formazan was measured at 570 nm with 680 nm as reference wavelength using a microplate reader (Infinite 200, Tecan). For data evaluation, background and reference wavelength corrected absorption values were averaged for the triplicates and expressed as "% cytotoxicity" referring to the untreated control containing only the solvent DMSO.

### LDH assay

A second set of cells was exposed to the test compounds for the LDH assay, which was performed using the Cytotoxicity Detection Kit LDH (Roche). For this assay, a positive control, leading to 100% cytotoxicity by lysing the cells completely, was included in the assay. The positive control was 2% Triton X-100 solution in the assay medium, as proposed by the manufacturer. After pre-incubation of the cells, before addition of the test compounds, the growth medium was exchanged from medium containing 5% FBS to medium containing only 1% FBS. Then, test compounds in DMSO were dosed and plates incubated as for the other assays for 24 h. For testing the released LDH activity, 100 μL of culture medium were transferred to a new 96 well plate. 100 μL of the reaction solution from the kit, containing the detection dye and the catalyst were then added and absorption was measured after 30 min at 490 nm with 655 nm as reference wavelength in an ELISA reader (Model 680, Biorad). As for the other assays, background values from wells without cells were subtracted and average values for the triplicates calculated. Cytotoxicity was then calculated according to the following equation: Cytotoxicity (%) = (experimental value - DMSO control)/(positive control - DMSO control) × 100.

The above described final test conditions were the result of preceding tests to optimize the assay described in the following: it was reported that FBS can lead to high background values in the LDH assay [8]. Therefore, its influence was first assessed comparing the normally used culture medium with 5% FBS to a reduced FBS concentration of 1%. This was done in presence of 2% Triton X-100. The positive control substance Triton X-100 has to be added, since the LDH assay does not detect the viable but only damaged or completely lysed cells.

### Combined alamarBlue^® ^and CFDA-AM assay

The fluorogenic indicator dyes alamarBlue^® ^(AB) (BioSource, Invitrogen) and CFDA-AM (5-carboxyfluorescein diacetate acetoxymethyl ester; Molecular Probes, Invitrogen) were used in combination on a third set of cells, since it was shown before that their fluorescent products can be detected at different non-interfering wavelengths [10]. AB was obtained as ready-to-use stock solution, which had to be diluted 200 times to obtain the working solution. CFDA-AM powder was dissolved in DMSO to a stock solution of 4 mM, which was diluted 1000 times to reach the 4 μM working solution. Working solutions were prepared in 1× Earle's-G medium [19], which was investigated in preceding experiments to support ZFL cell viability during the assay incubation period. For this purpose, cells were exposed to the dyes dissolved in culture medium without FBS or in Earle's-G medium. Since Earle's-G medium showed the same good correlations between cell number and fluorescence and lower background values it was chosen for standard test conditions. The AB/CFDA-AM assay was then performed according to [10]. In brief, after 24 h of incubation with the test compounds, the medium was aspirated off completely and 100 μL/well of the alamarBlue/CFDA-AM working solution were added. After 30 min of incubation, fluorescence was measured at 530 nm excitation/590 nm emission for AB and 485 nm/535 nm for CFDA-AM, respectively, using a fluorescence plate reader (Infinite 200, Tecan). For data evaluation, average values of triplicates were calculated after background correction and all average values for the treatments referred to the DMSO control, in order to express data as "% cytotoxicity".

### Data evaluation and statistical analysis

For each single assay, data were evaluated separately for three independent experiments. Dose-response curves were fitted using a nonlinear-regression sigmoidal dose-response curve model provided in the GraphPad Prism software (GraphPad software, Inc., San Diego, USA). EC_10 _and EC_50 _values were derived from these fitted curves for the single experiments. Final EC-values (Table 1) were calculated as average of three independent experiments with the standard deviation of the mean indicating the variation. Data from the different assays were statistically compared using One way ANOVA analysis with Tukey's test as post-ANOVA analysis (p < 0.05).

Intra-assay variabilities were calculated as coefficient of variance (CV), based on the triplicate values within each individual experiment. In a second step, CV values for the three individual experiments for each tested compound were determined calculating the median CV in a low (0–20%) and high (80–100%) cytotoxicity range. The inter-assay variability was assessed based on the EC_10 _and EC_50 _values. Median CV values were calculated using the EC values from each three individual experiments for each tested compound. Signal-to-noise ratios were calculated based on Tam exposure experiments for each compound concentration tested. Average absorption or fluorescence signals from wells with differently treated cells were divided by the average signal from wells containing only the test medium without cells, representing the background values. Then, median values for the low (0–20%) and high (80–100%) cytotoxicity range were calculated.

## Abbreviations

4-OHT: 4-Hydroxy-Tamoxifen; AB: alamarBlue^®^; BaP: Benzo[a]pyrene; CFDA-AM: 5-carboxyfluorescein diacetate acetoxymethyl ester; DMEM: Dulbecco's modified Eagle's medium; DMSO: dimethyl sulfoxide; FBS: fetal bovine serum; Flus: Flusilazole; HEPES: 4-(2-Hydroxyethyl)-1-piperazineethanesulfonic acid; INT: 2-[4-iodophenyl]-3-[4-nitrophenyl]-5-phenyltetrazolium chloride; LDH: lactate dehydrogenase; MTT: 3-[4,5-dimethylthiazol-2-yl]-2,5-diphenyl tetrazolium bromide; Tam: Tamoxifen.

## Authors' contributions

SKB carried out the experimental work. SKB and TL together conceived of the study and drafted the manuscript. Both authors read and approved the final manuscript.
